# Report of the Proceedings of the Forty-Fourth Annual Meeting of the Michigan State Dental Association

**Published:** 1900-07-15

**Authors:** 


### DENTAL REGISTER.

Vol. LIV. JULY 15, 1900. No. 7.

### REPORT OF THE PROCEEDINGS OF THE FORTY-FOURTH ANNUAL MEETING OF THE MICHIGAN STATE DENTAL ASSOCIATION.

The first session of the Michigan State Dental Association meeting for the year 1900 was called to order by the president, Henry C. Raymond, at 2:15 P.M., Monday, June 11th, at Kalamazoo. The meeting was opened with prayer by the Rev. Mr. McLaughlin, of Kalamazoo. The president then called upon Dr. Westbrook, who had been asked by the mayor of Kalamazoo to give the address of welcome.

DR. WESTBROOK: Mr. President, Ladies and Gentlemen—The mayor was obliged to leave the city this morning; I did my best to persuade him to remain, but he requested me to be here and welcome you. Whether it was through fear, or modesty, or diffidence, that he left, I am unable to say, nevertheless he desired me to extend to you a hearty welcome to our city. He had nothing to fear, so far as I am able to see, for you seem to be a quite orderly crowd. You, also, have nothing to fear, as I have not seen a policeman around this afternoon; at noon I saw them gathered in a body—to protect themselves, I suppose. They were asking what fine-looking lot of men these were coming into our city? Some one told them they were dentists, and that each one had a pair of forceps up his sleeve and was ready to use them. The last seen of the policemen they were making for the woods as fast as they could.

As I have no key to the city I can not give you same, but everything in the city is wide open, and I extend to you the freedom of the city. Enjoy yourselves; we know you will behave yourselves. I extend to you the heartiest welcome, and hope each one will take away something which will be of great benefit to him in the future.

The president then called upon Dr. A. T. Metcalf, of Battle Creek, to give the response.

Dr. Metcalf: Mr. President, Ladies and Gentlemen, and Dr. Westbrook, who represents the mayor of the city here to-day—I want to say to you it gives me a great deal of pleasure to have the honor to make this response. How it comes about I do not know, unless it be through the innate modesty of our president, or that some committee has put up a job on him and delegated the honor to me.

I was a practicing dentist in this city almost a half century ago, and I do not know but that I am the oldest dentist here to-day who was present at the organization of the State Dental Association; it is possible there may be one or two living, but certainly they are not present to-day. I do not think, however, there is one in the State. At that meeting there were only eight or ten of the dentists of the State present; it was away back in 1856, in Detroit. From that has sprung this association of dentists, through whose efforts all the leading practitioners are brought together; and, taking them as a class, they are the equal of the dentists of any State in the Union. I believe, too, that this Association was one of the first formed in the United States.

When I lived in Kalamazoo your beautiful park was in a condition of nature and there was a rail fence about it. It is now quite nice, and has a fine fountain—not beautiful, but it is large and answers the purpose very well. The waterworks of your city at one time was one of the wonders of the world. A well was built upon the top of the ground, and then sunk down thirty or forty feet into the ground, and from that place the city gets its water supply. However, it has been fortified by other wells. For ten or twelve years I think it was the finest water in the world. I always come back here to get a drink, although we have good water in Battle Creek—for sprinkling streets, fire, etc.

Your honor, this has been a remarkable city for many things. It was in this city that the first woman's club of the country, I believe, was organized. The late Mrs. S. K. Stone was first to set the matter moving. The ladies of this city are the most intelligent in the State. Yours is one of the best governed cities in the State of Michigan; it does not owe a dollar of indebtedness. Battle Creek is very well off, but we owe money.

There is one thing, your honor, that I want to speak of in relation to the city of Kalamazoo: The dentists of this city have done something for the profession. In this city was manufactured and placed upon the market the first dental engine that was ever made. Here, also, was manufactured the first annealing lamp, and here was invented and manufactured the first practical automatic mallet; it may not have been the first, but it was a decided improvement on all predecessors.

It is here, too, that the electric railway was invented, and where the first one that was ever made was built and put into experimental operation. Not on the streets of the city but in the yard of the man who invented it, and it was on exhibition here for two or three years before any street railway was built. It has been proven in the courts that this invention was made in this city by George F. Greene, who died a few years ago.

I noticed on coming out on the cars to-day, in looking over this program or notice which was sent out by the dentists of this city, that you are to greet us with "great joy knawing at the hinges on the back door of your souls."

Well, we are here to relieve this knawing on your hinges, or the back doors of your souls, and the first opportunity you give us to apply the ointment we shall most cordially embrace.

This is one of the most beautiful cities of the State of Michigan. We have only to walk about its streets to be convinced of that fact, the fine homes and every thing that goes to make up home life you will find here. When you get a little off in your upper works this is the best place in the world to come for help, that most beautiful spot on the hill, which is cared for by the State of Michigan. It is one of the best managed institutions of its kind in the United States. I am so well acquainted with the institution and with the men who are at the head that I have been trying for two or three years to make the officers of our county recognize the fact that I was wrong in the upper story, so I could come here to live. I shall very soon be in a fit condition, and perhaps you think I am now.

The hospitality of the people of this city is proverbial, and I know when we get through with this meeting and go to our homes we can say truly and emphatically that we have enjoyed our meeting here because of the kind hospitality of your citizens.

---

The Secretary read an invitation from the Superintendent of the Asylum for Insane for the dentists to visit them at their convenience.

On motion the invitation which had been extended to visit the asylum was accepted with thanks, and the Executive Board were ordered to make suitable arrangements to visit the asylum.

The President read his annual address.

